# Clinical Efficacy Analysis of the Personalization of Prosthetic Abutments in Implant Supported Restorations in Comparison to Available Standard Titanium Abutments

**DOI:** 10.3390/jpm13091402

**Published:** 2023-09-19

**Authors:** Magdalena Eugenia Obădan, Ioana Mitruț, Mihaela Ionescu, Florian Obădan, Daniel Adrian Târtea, Marcel Adrian Popescu, Sanda Mihaela Popescu, Andreea Maria Smarandache, Horia Octavian Manolea

**Affiliations:** 1 Department of Dental Materials, University of Medicine and Pharmacy of Craiova, 200349 Craiova, Romania; 2 Department of Medical Informatics and Biostatistics, University of Medicine and Pharmacy of Craiova, 200349 Craiova, Romania; 3 Department of Oral Rehabilitation, University of Medicine and Pharmacy of Craiova, 200349 Craiova, Romania; 4 Department of Dental Prosthetics, University of Medicine and Pharmacy Carol Davila, 050474 Bucharest, Romania

**Keywords:** dental implant, customized abutment, prosthetic restoration

## Abstract

Personalized medicine has become an important direction to offer better solutions for health problems. In implantology, this trend was materialized through customizing dental abutments to each clinical situation. The demands for better esthetics and function of implant-supported restorations have imposed a more personalized variety of prosthetic abutments. This retrospective study compared clinical efficiency of personalized implant abutments with standard implant abutments in multiple implant restorations. Clinical data of patients who were admitted in a private clinic between 2011 and 2022 and received dental implant treatments were collected. All complications and undesired events from the patients’ medical record charts were statistically analyzed. The implants were loaded using either standard or customized abutments. For complete arch rehabilitations with the SKY Fast & Fixed protocol, standard titanium prosthetic abutments were used. Our results suggest that the abutments choice for patients has moved throughout the years more towards the use of customized abutments. The number of customized abutments (414) was higher compared with the number of standard abutments (293). In our database, the most used abutments for the anterior area implants were made of titanium and zirconia, whereas for the posterior area, the preferred abutments were mostly titanium. The standard abutments were used almost entirely for immediate loading and implantation in both anterior and posterior areas (Fast & Fixed protocol). Complications were encountered mainly in restorations with standard abutments (9.22%) compared to customized abutments (2.7%), with titanium abutments being the most reliable, having only 1.79% complications.

## 1. Introduction

Personalized medicine has become an important approach to offering better solutions for health problems. In implantology, this trend was materialized through the use of customized dental abutments tailored to each clinical situation. In the present times, there is a growing demand for improved aesthetics and functionality in implant-supported crowns, leading to a preference for a more personalized variety of prosthetic abutments. In fact, some authors consider that dentistry cannot be imagined without them [1].

Customized abutments were first used by Dumbrigue in 2002 [2] and offer several advantages compared to standard prefabricated ones. These advantages include better support of soft tissues, reduced cement quantity in the peri-implant sulcus [1,3], and platform switching, which results in less crestal bone loss [4,5,6]. Additionally, the choice of a suitable cement is important, as it should reflect the possibility of being detectable (radiopacity), water soluble, easily removable, antibacterial, and anti-inflammatory [7,8].

Abutment designs may be customized to enhance the emergence profile of the crown in relation to the soft tissue or to increase crown retention [9].

The aim of the current study is to compare the clinical efficiency of personalized implant abutments with standard implant abutments in terms of their frequency of use and the occurrence of complications and unwanted events.

Thus, we have conducted comparisons in the following areas:

(a) Prosthetic solutions used with standard and customized abutments divided by type of materials;
(b) Incidence of complications;
(c) The evolution of dental implants in immediate loading vs. delayed loading scenarios.

## 2. Materials and Methods

This retrospective study was conducted following the STROBE criteria and analyzed the patients’ data who were admitted to a private clinic (Implant Consult Clinic) and received implant-supported prosthetic restorations between 2011 and 2022, with varying follow-up periods.

The patients received dental implant treatments starting from the year 2011. The study strictly followed the European Union’s General Data Protection Regulation on patient data protection and discretion (GDPR) [10] and the 1975–2003 Declaration of Helsinki [11]. Moreover, it was approved by the Ethics Committee of the University of Medicine and Pharmacy of Craiova (No. 63/19.04.2022).

The inclusion criteria consisted of clinically healthy patients ASA 1 & 2 [12] with a medium level of oral hygiene and treated with blueSKY and copaSKY from SKY dental implants (Bredent medical GmbH, Senden, Germany) with either immediate or delayed loading.

For single-tooth restorations, immediate loading was performed using standard “elegance abutments,” which had a titanium base and ceramic-reinforced PEEK (polyetheretherketone) composite material. For multiple teeth restorations, customized titanium and zirconia abutments were also used. For bigger restorations, especially for complete arch rehabilitations, we chose the SKY Fast & Fixed protocol, for which standard titanium prosthetic abutments were used. This method allowed occlusal screw retention of a PMMA bridge extending from the second premolar to the other second premolar.

All complications and undesired events were noted in the patients’ medical record folders.

The exclusion criteria encompassed patients treated with single-piece implants, patients who did not return for the recall sessions, patients with a low level of oral hygiene, and patients with uncontrolled systemic diseases.

For each patient, the following data were recorded: age, sex, position of the implants (posterior or anterior area), abutment type, date of implantation, date of loading, complications, and date of definitive restoration.

We named the abutments as follows: S (standard titanium abutment), and the 3 customized abutments as T (titanium abutments), Z (zirconia abutments), and P (PEEK abutments).

The data were initially grouped using Microsoft Excel (San Francisco, CA, USA). For the descriptive analysis, continuous variables were expressed in terms of absolute and relative frequencies (%) and mean ± standard deviation (SD). The analysis of the correlations between these variables was performed using the Statistical Package for Social Sciences (SPSS), version 20 (IBM Corp., New York, NY, USA), and the following statistical tests: Kendall’s tau-b correlation, Shapiro–Wilk’s test for data normality analysis, Levene’s test of equality of variances, Mann–Whitney U test, and Kruskal–Wallis H test (followed by Dunn’s (1964) procedure with a Bonferroni correction for multiple comparisons when necessary). The chi-square test (association, independence, or homogeneity) was used for categorical data. For the purposes of the present study, the significance level was set at *p* < 0.05, with a confidence interval (CI) of 95%.

## 3. Results

**Figure 1:**
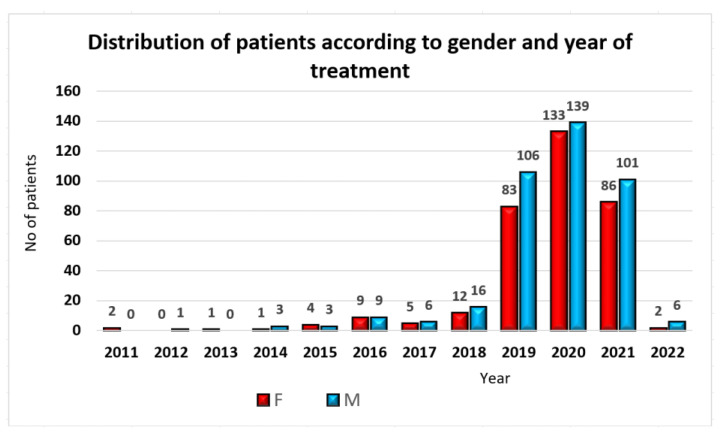
Patients’ distribution by gender and year of treatment.

The initial study involved 728 patients, comprising 338 females (representing 46.43% of the entire study lot) and 390 males (53.57%), with ages between 19 and 82 years old (mean age 52.69 ± 11.68) (Figure 1).

The distribution of patients by age and gender is indicated in Table 1.

Half of the patients included in the study (364 patients, 50%) received implants in the posterior area only, while 72 patients were treated with implants in the anterior area (72 patients, 9.89%) only. A number of 292 patients (40.11%) were treated with implants in both the anterior and posterior areas. A chi-square test of homogeneity was conducted to examine the relationship between gender and implant area. All expected cell counts were greater than or equal to five, with the lowest expected cell count being equal to 33.4. Males received more implants in the anterior area (n = 50, 12.8% vs. n = 22, 6.5%) and both the anterior and posterior areas (n = 163, 41.8% vs. n = 129, 38.2%) compared to females. Conversely, females received more implants in the posterior area (n = 187, 55.3% vs. n = 177, 45.4%). The two multinomial probability distributions were not equal in the population, showing a statistically significant difference, χ^2^(2) = 11.467, *p* = 0.003.

For 21 patients (representing 2.88% of the entire study population, comprising eight females and thirteen males), the abutments were never loaded due to not showing up for the visits. The loading procedure was completed for 707 patients (representing 97.12% of the entire study population, consisting of 330 females and 377 males).

**Figure 2:**
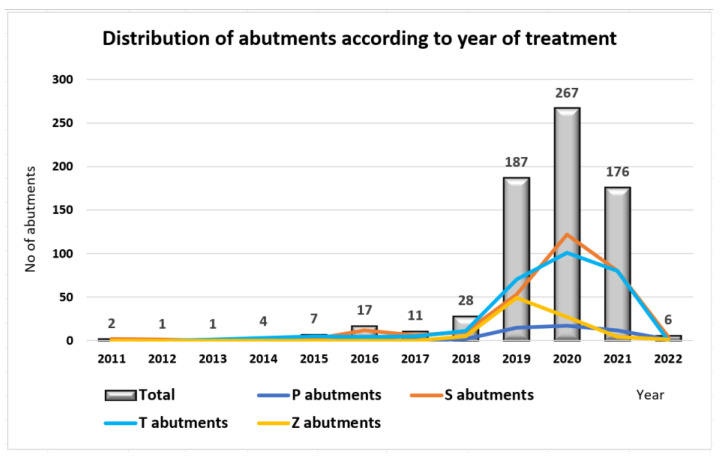
Abutments’ distribution by year of treatment.

**Figure 3:**
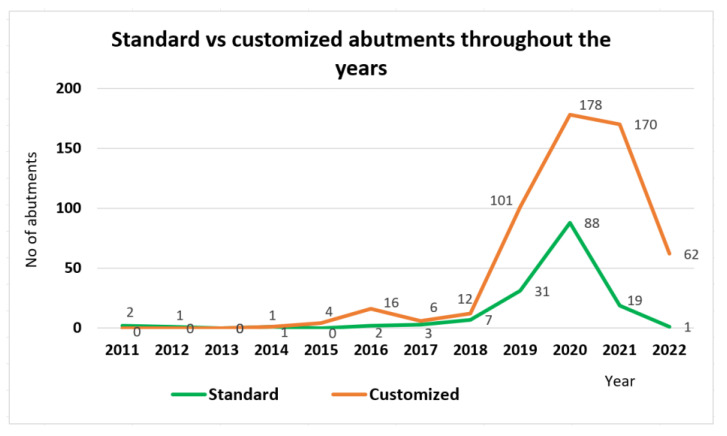
Standard vs. customized abutments throughout the years.

The acquired data analysis has shown that the abutments choice has moved more towards the usage of customized abutments (Figure 2 and Figure 3). While in 2011−2013, the abutments were 100% standard, by 2020−2022, the percentage of standard abutments had significantly decreased compared to customized ones.

**Figure 4:**
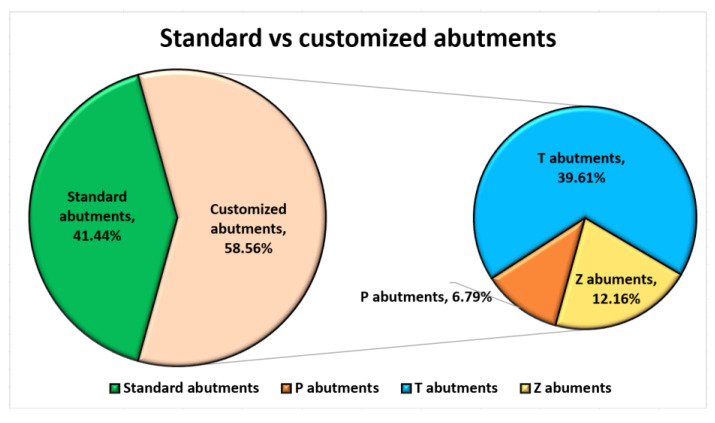
Distribution of standard vs. customized abutments used.

The analysis of the abutments received by patients, according to our classification of standard implants and customized abutments, were 58.56% and 41.44%, respectively. The customized abutments consisted of titanium customized abutments (39.60%), Zirconia abutments (12.16%), with the lowest number represented by ceramic-reinforced PEEK abutments (6.79%) (Figure 4).

BioHpp PEEK abutments (n = 30, 62.5% vs. n = 18, 37.5%), standard abutments (n = 167, 57.0% vs. n = 126, 43.5%), and customized titanium abutments (n = 141, 50.4% vs. n = 139, 49.6%) were more frequently chosen by males than females. In contrast, zirconia abutments (n = 55, 51.4% vs. n = 52, 48.6%) were mainly selected by females. However, there were no statistically significant differences in abutment type regarding gender, χ^2^(3) = 6.399, *p* = 0.094 (Table 2).

A Kruskal–Wallis H test was run to determine if there were differences in the age of recipients of the four types of abutments: P (n = 48), S (n = 293), T (n = 280), and Z (n = 86). Distributions of ages were similar for all groups, as assessed by the visual inspection of a boxplot. However, median ages were statistically significantly different between the groups, χ^2^(3) = 59.841, *p* < 0.005. Pairwise comparisons were performed using Dunn’s (1964) test with a Bonferroni correction for multiple comparisons. Statistical significance was accepted at the *p* < 0.0083 level. This post hoc analysis revealed statistically significant differences in ages between the Z (rank 45 years old) and T types (rank 51 years old) (*p* = 0.002), Z and S types (rank 56 years old) (*p* < 0.005), and T and S types (*p* < 0.005). However, no significant age differences were observed between the P type (rank 51 years old) and the other types or between any other combinations of groups (Table 2).

A chi-square test of independence was conducted to examine the association between abutment type and implantation area. All expected cell frequencies were greater than five. There was a statistically significant association between abutment and area, χ^2^(6) = 669.217, *p* < 0.0005. This association was moderately strong, with a Cramer’s V = 0.688 (Table 2).

**Figure 5:**
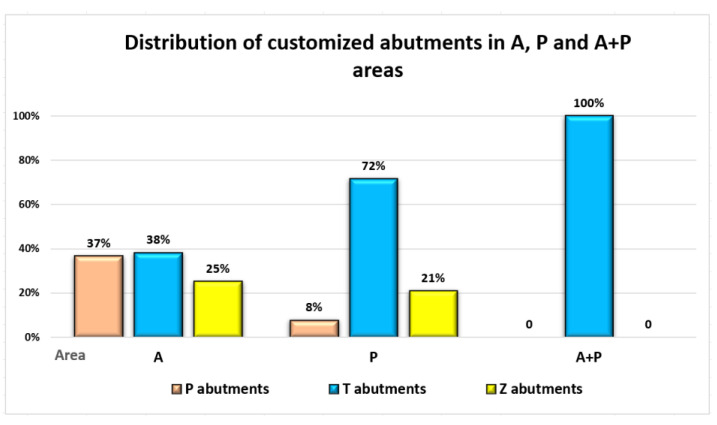
Distribution of customized implant abutments in A, P and A + P areas.

Concerning the customized abutments’ use based on the implant position area, a higher number of T abutments were utilized in the posterior area, while in the anterior area, both P and T abutments were mostly used (Figure 5).

**Figure 6:**
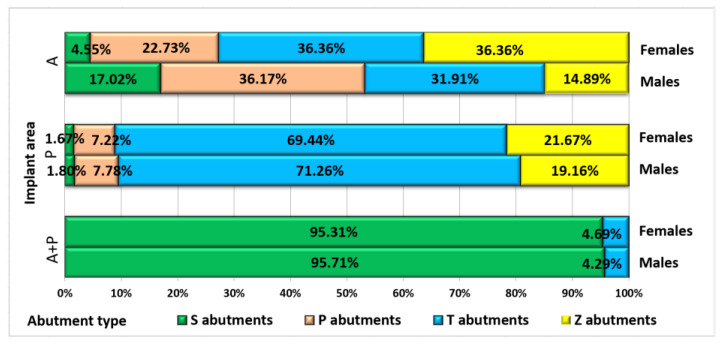
Patients’ distribution by gender, implant area and abutment type.

In cases where patients received implants both in anterior and posterior areas, particularly for long bridges using the Fast and Fixed method, the use of standard titanium abutments prevailed (Figure 6).

### 3.1. Immediate/Delayed Loading

**Figure 7:**
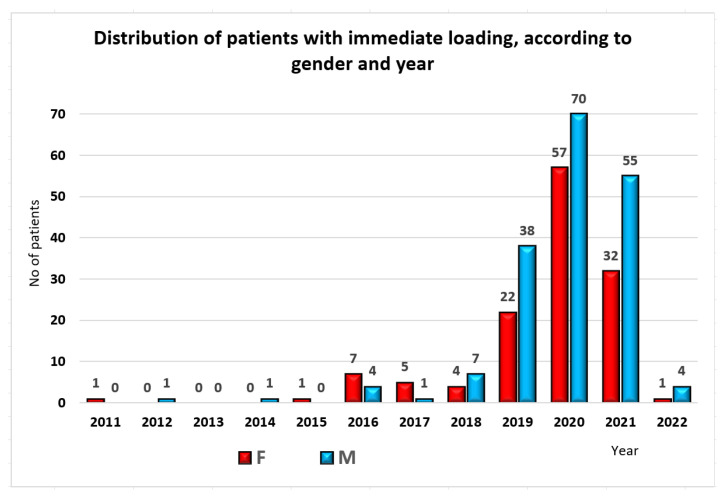
Distribution of patients with immediate loading, by gender and year of treatment.

Less than half of the patients (311, representing 43.99%) were treated with immediate loading, mostly males (n = 182, 58.33% vs. n = 130, 41.67%). A chi-square test for association was conducted to examine the relationship between gender and for implant immediate loading. All expected cell frequencies were greater than five. There was a statistically significant association between gender and immediate loading option, χ^2^(1) = 5.630, *p* = 0.018 (Figure 7).

Patients with immediate loading received mostly standard abutments (n = 284, 96.9% vs. n = 9, 3.1%) compared to patients with delayed loading (Table 3). The remaining 10% of patients with immediate loading received the following abutments: titanium (n = 19, 6.1%), PEEK (n = 7, 2.2%), and zirconia (n = 2, 0.6%). There was a statistically significant association between abutment type and immediate loading option, χ^2^(3) = 567.611, *p* < 0.0005. This association was strong, with a Cramer’s V = 0.896.

Regarding the area distribution, patients with immediate loading received mostly a full arch restoration in A + P area (n = 273, 87.5% vs. n = 18, 4.6%) compared to patients without immediate loading. The remaining 12.5% of patients with immediate loading received restorations in the following areas: anterior (n = 33, 10.6%) and posterior (n = 6, 1.9%). There was a statistically significant association observed between implantation area and immediate loading option, χ^2^(2) = 544.763, *p* < 0.0005. This association was strong, with a Cramer’s V = 0.878.

**Figure 8:**
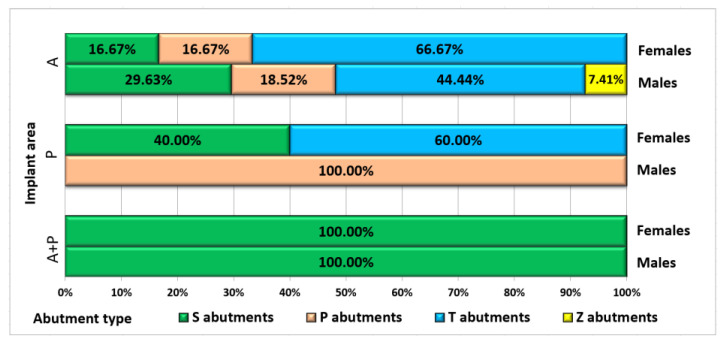
Distribution of patients with immediate loading, by gender, implant area and abutment type.

The distribution of patients with immediate loading, categorized by gender, implant area and abutment type, is emphasized in Figure 8.

### 3.2. Complications

From the total of 707 patients for whom the loading procedure was completed, 5.09% (36 patients) suffered various complications with the abutments, 16 females (44.44%) and 20 males (55.56%), with ages ranging from 37 to 79 years (mean value 57.58 ± 8.53). Three quarters of these abutments were standard (27, representing 9.22% of all standard abutments), and only one quarter were customized (nine, representing 2.17% of all customized abutments).

Prosthetic fractures were the most common type of accident, recorded for 21 patients (58.33% from all patients with accidents), followed by explantation for nine patients (25%), decementation in three patients (8.33%), loosened screw in two patients (5.56%) and fistula in one patient (2.78%) (Table 4).

For further analysis, accidents were divided into three categories: prosthetic fractures (58.33%), explantation (25%), and other complications (six patients, 16.67%).

More than 80% of accidents happened for patients with immediate loading (30 patients, 83.33%), while 16.7% of accidents (six patients, 16.67%) happened for patients with delayed loading. A chi-square test for association was conducted between immediate loading and accidents. All expected cell frequencies were greater than five. There was a statistically significant association between immediate loading and accidents, χ^2^(1) = 23.644, *p* < 0.0005, with a weak association between those parameters, φ = 0.183, *p* < 0.0005 (Table 4).

A Kruskal–Wallis H test was run to determine if there were differences in age between the three categories: fracture (n = 21), explantation (n = 9) and other (n = 6). Distributions of ages were similar for all groups, as assessed by visual inspection of a boxplot. Median ages (58 years old for patients with fractures and explantation, 59 years old for other accidents) were not statistically significantly different between groups, χ^2^(2) = 0.162, *p* = 0.922 (Table 4).

The distribution of patients according to gender and accident type is indicated in Table 4. Males experienced more fractures (n = 13, 61.9% vs. n = 8, 38.1%) and more explantations (n = 5, 55.6% vs. n = 4, 44.4%) than females, whereas females experienced more of the "other" category of complications (n = 4, 66.7% vs. n = 2, 33.3%). However, there were no statistically significant differences in the type of complication regarding gender, χ^2^(2) = 1.543, *p* = 0.462.

Regarding abutment types, 75% of accidents (27) happened for type S, 13.89% (five accidents) happened for type T, and 5.56% (two accidents each) with types P and Z (Table 4). Therefore, there were statistically significant differences in abutment type regarding complication categories, χ^2^(6) = 24.174, *p* < 0.0005, with 10 cells having expected count less than five.

Regarding the implantation area, 72.22% of complications (26 cases) happened for A + P area, 13.89% (five cases) happened in the posterior area, and 13.89% (five cases) occurred for A area (Table 4). Therefore, there were statistically significant differences in the implantation area regarding complications, χ^2^(6) = 24.769, *p* < 0.0005, with seven cells having expected count less than five.

## 4. Discussion

In implantology nowadays, the prosthetic phase has shown a growing inclination towards the utilization of customized abutment [13]. Our study has revealed that in recent years, customized abutments have become more frequently chosen. This evolution was possible due to numerous factors, such as the availability of new equipment and partnerships that have made the use of customized abutments more accessible for dentists in clinical practice. 

In our database, the most used abutments for the anterior area implants were made of titanium and zirconia, whereas for the posterior area the abutments preferred were mostly titanium. The standard abutments were used almost entirely on implants used in both anterior and posterior regions, typically following the Fast & Fixed protocol.

The availability of new equipment and partnerships that have made the use of customized abutments more accessible for dentists in clinical practice, especially when esthetics play an important role. Anterior implant sites are often characterized by a high scalloped mucosal margin with a distance of up to 7–8 mm to the implant shoulder. Customized abutments enable the creation of an individualized emergence profile for the restoration, simplifying the removal of excess cement compared to standardized abutments [14]. Customized titanium abutments were used more in the posterior region and exhibited only five complications (1.79%).

Today, a large variety of biocompatible materials are available due to the widespread use of CAD/CAM technology. Studies have shown that both titanium and zirconia have excellent cell adhesion properties [15,16]. CAD/CAM customized abutments have been proven to have a good aesthetic and functional prognosis of implant-supported restorations. Moreover, they contribute to enhancing the final shape of prostheses, improving the stability of implant-supported prostheses, and efficiently transmitting masticatory forces to the implant [13].

Different factors are crucial for making the right decision regarding the optimal material and reconstruction type for the posterior region. For implant reconstructions, irrespective of their location, an adequate emergence profile is a prerequisite for healthy soft and hard tissue integration (biologic width), as well as for the ease of cleaning for the patient and achieving a natural appearance. In molar areas, a large deviation between implant and crown diameters can be found. In these situations, customized abutments, together with the ideal emergence profile, allow the crown margin to follow the present mucosal outline [17].

With increased soft tissue thickness (>2 mm), it can be expected that the abutment material will have less influence on the soft tissue color [18,19].

Some studies have shown that two-piece zirconia abutments, which consist of a prefabricated titanium base bonded to a zirconia abutment, are to be preferred over one-piece zirconia abutments because of their higher fracture resistance [20,21,22,23].

The posterior region of the jaw bears a higher load and as such must be mechanically stable. Studies have shown that while titanium abutments remain stable on the implant without changing their structure, zirconia abutments under cyclic loading manifest structural changes and a greater rate of wear. The contact between abutment and implant can produce micromotion during the application of cyclic loads, and because zirconia abutments are harder than titanium implants, they can affect the structure of the implant hexagon [24,25]. Due to the above-listed challenges associated with the use of zirconia abutments, they are limited to single and short-span restorations in anterior and posterior regions but are avoided in long-span and full-arch restorations in the A + P regions.

The microgap at the implant-abutment connection is obviously important for crestal bone loss progression. If it is located close to the bone level, it might cause bone resorption in flat-to-flat connections. Therefore, implants with platform switching, in which the microgap is shifted inward horizontally away from the bone crest, are advised because ingress of oral fluid and bacteria can be avoided. In addition to that, the stability of the implant-abutment connection seems to be important, especially if the implant is placed subcrestally. While connection stability is not an ultimate factor, as low levels of bone loss have been reported around implants with a simple internal hexagon. However, the subcrestal implant position relates to a different environment, possibly more sensitive to any micro-movements of the abutment connection; thus, the need for a stable junction is more relevant [26].

An optimized occlusal load transfer through prosthetic and implant components to the bone-implant interface is a key factor in implant prognosis, especially for implants subjected to high biomechanical forces, i.e., single molar implants. This type of prosthetic abutment significantly influences the bone stress/strain in immediately loaded implants [27].

Zirconia abutments are considered to potentially damage the titanium of the implant’s connection due to their hardness [28]. In addition, researchers have demonstrated that zirconia abutments can influence the appearance of cracks and microfractures that most likely occur during the shift of the abutment accommodation against the implant platform [25]. In the current study, the decementation of two zirconia abutments (2.33%) was the only complication observed.

Generally, immediately loaded implants exhibit higher values of bone stress and strain than delayed loaded implants, where the implants are considered osseointegrated [29]. The current research reinforces this point because there were more complications in the immediate loaded implant restorations. However, this may be due to the fact that 93.8% of the immediate loaded implants in this paper have longer spans since they were mostly Fast & Fixed cases used in the A + P region, thereby being exposed to more stress.

In the data collected in our study, the implants with immediate loading received mostly standard titanium abutments. Concerning customized abutments, they were mostly used in delayed loading. As far as materials used for abutments, in the personalized group, titanium is still the most used material because of its characteristics, although more esthetic materials like zirconia and PEEK have started to gain attention [30,31,32].

Another aspect that has been demonstrated to affect the preservation of marginal bone is the stability of the soft tissue around the implant–abutment interface [33]. In fact, Piatelli et al. have shown how the interactions between cellular components and implant–abutment materials influence the stages of the healing process around implants [34]. The quality of the soft tissue was not observed in the current paper and is an area for further research.

The clinical success of the implants and the stability of the abutment/implant interface are influenced by several factors, such as the material of the abutments, the adjustment and precision in the fabrication of its components, its contamination by saliva, the preload on the retaining screw, the microgap, the connection geometry, and aging [35,36,37,38].

PEEK abutments have been introduced into implant dentistry as a viable alternative to current implant abutment materials [39]. Studies have indicated, firstly, that PEEK abutments should be used as a temporary abutment material [40,41]. Saravi in 2022 concluded that the application of PEEK can be used as a novel definitive implant abutment material, as PEEK abutments showed superior load-bearing properties compared to zirconia, although it was associated with greater microgaps at the implant–abutment complex [39].

In our database, the patients received ceramic-reinforced PEEK abutments mostly on immediately loaded implants and, in general, more in the anterior area with few complications. PEEK abutments were not used for final restorations.

## 5. Conclusions

Prosthetic customized abutments are used more often nowadays for multiple implant restorations. Our study has shown that customized abutments have a lower rate of complications and are mostly used in delayed loading. In the personalized abutment restorations group, titanium was the most used material. It is important for clinicians to have vast knowledge and be updated with all the information regarding customized abutments, so that the best solution can be chosen for the patient. Seeking advanced materials and improved geometries can lead to a better therapeutical option for patients in the future. As each patient has their own morphological and functional particularities, we can conclude that the future belongs to customization regardless of the type of abutment and prosthetic material, be it titanium, zirconia, or ceramic-reinforced PEEK.

## Figures and Tables

**Table 1:** Patients’ distribution by decade and gender.

Gender	Age (Years Old)						Total
	19−29	30−39	40−49	50−59	60−69	70−82	
Females	11	39	96	94	77	21	338
Males	6	38	102	115	103	26	390
Total	17	77	198	209	180	47	728

**Table 2:** Abutment distribution by gender and implantation area.

Group	n/%	Standard	P abutment	T abutment	Z Abutment	*p*
Females	330 (100)	126 (38.2)	18 (5.5)	139 (42.1)	47 (14.2)	0.094 *
Males	377 (100)	167 (44.3)	30 (8.0)	141 (37.4)	39 (10.3)	
Age (mean ± SD)	52.69 ± 11.68	56.54 ± 9.88	50.64 ± 11.34	51.60 ± 11.79	45.43 ± 12.81	<0.0005 **
A	69 (100)	9 (13.0)	22 (31.9)	23 (33.3)	15 (21.7)	
P	347 (100)	6 (1.7)	26 (7.5)	244 (70.3)	71 (20.5)	<0.0005 *
A + P	291 (100)	278 (95.5)	0 (0.0)	13 (4.5)	1 (0.3)	

* Chi-Square test. ** Kruskal–Wallis test.

**Table 3:** Loading type distribution by abutment type and implantation area.

Group	Loading			*p* *
	Delayed	Immediate	Total	
Standard	9 (3.1%)	284 (96.9%)	293 (100%)	<0.0005
Customized	386 (93.2%)	28 (9.6%)	414 (100%)	
P abutment	41 (85.4%)	7 (14.6%)	48 (100%)	
T abutment	261 (93.2%)	19 (6.8%)	280 (100%)	
Z abutment	84 (97.7%)	2 (2.3%)	86 (100%)	
A	36 (52.2%)	33 (47.8%)	69 (100%)	<0.0005
P	341 (98.3%)	6 (1.7%)	347 (100%)	
A + P	18 (6.2%)	273 (93.8%)	291 (100%)	

* Chi-Square test.

**Table 4:** Patients’ distribution by gender, abutment type, area, and accident type.

Group	Total (N)	Total Complication (n, % from N)	Fractures	Explantation	Decementation	Loosened Screw	Fistulae	*p*
Females	330	16 (4.85%)	8	4	1	2	1	0.462 *
Males	377	20 (5.31%)	13	5	2	-	-	
Age (mean ± SD)	707	36 (5.09%)	58.24 ± 9.46	55.55 ± 8.23	57.33 ± 4.72	55.50 ± 4.95	-	0.922 **
Standard	293	27 (9.22%)	19	7	-	1	-	<0.0005 *
Customized	414	9 (2.17%)	2	2	3	1	1	
P	48	2 (4.17%)	-	-	1	-	1	
T	280	5 (1.79%)	2	2	-	1	-	
Z	86	2 (2.33%)	-	-	2	-	-	
A	69	5 (7.25%)	-	3	1	-	1	<0.0005 *
P	347	5 (1.44%)	-	2	2	1	-	
A + P	291	26 (8.93%)	21	4	-	1	-	
Delayed loading	396	6 (1.52%)	2	1	3	-	-	<0.0005 *
Immediate loading	311	30 (9.65%)	19	8	-	2	1	

* Chi-Square test. ** Kruskal-Wallis test.

## Data Availability

The authors declare that the data of this research are available from the correspondence authors upon reasonable request.
